# DEMETRA: An ACT-Based Virtual Coach to Support Healthier Lifestyles in Overweight Pregnant Women—Protocol for a Feasibility Pilot Study

**DOI:** 10.3390/ijerph23040483

**Published:** 2026-04-11

**Authors:** Anna Elena Nicoletti, Barbara Purin, Silvia Rizzi, Carlo Dalmonego, Anna Bezzeccheri, Silvia Corradini, Stefania Poggianella, Claudia Paoli, Barbara Burlon, Marina Zorzi, Cecilia Lazzari, Stefania Depaoli, Ornella Fronza, Enrica Lorenzato, Debora Marroni, Stefano Forti, Fabrizio Taddei

**Affiliations:** 1 Digital Health Research, Centre for Digital Health & Wellbeing, Fondazione Bruno Kessler, Via Sommarive 18, 38123 Trento, Italy; purin@fbk.eu (B.P.); srizzi@fbk.eu (S.R.); carlo.dalmonego@gmail.com (C.D.); anna.bezzeccheri@gmail.com (A.B.); silvia.corradini85@gmail.com (S.C.); spoggianella@fbk.eu (S.P.); forti@fbk.eu (S.F.); 2 Transmural Obstetric Gynecological Department, Healthcare Trust of the Autonomous Province of Trento (APSS), 38122 Trento, Italy; claudia.paoli@apss.tn.it (C.P.); barbara.burlon@apss.tn.it (B.B.); marina.zorzi@apss.tn.it (M.Z.); cecilia.lazzari@apss.tn.it (C.L.); stefania.depaoli@apss.tn.it (S.D.); ornella.fronza@apss.tn.it (O.F.); enrica.lorenzato@apss.tn.it (E.L.); fabrizio.taddei@apss.tn.it (F.T.); 3 Operating Unit of Psychology, Healthcare Trust of the Autonomous Province of Trento (APSS), 38122 Trento, Italy; debora.marroni@apss.tn.it

**Keywords:** pregnancy, overweight, healthy eating, healthy lifestyles, mental well-being, ACT, eHealth, digital intervention, usability, user-centred design

## Abstract

During pregnancy, women are more inclined to modify their habits and lifestyle to find a new balance and promote well-being for themselves and the child-to-be. However, the availability of nutritional and psychological support is often limited by stigma, geographic barriers, and a lack of services. Digital health tools are emerging as possible solutions to cover these needs. This study explores the acceptability, feasibility, and user experience of Demetra, a virtual coach based on Acceptance and Commitment Therapy (ACT), designed to promote healthy lifestyles and mental well-being. Fifty pregnant women will be enrolled in the feasibility study of the intervention. It starts with an educational part on the foundations of healthy eating and suggestions about lifestyle habits, followed by a six-week psychoeducational module. Content is delivered through text, audio, and video formats. User experience and engagement will be measured through validated questionnaires and semi-structured interviews. Psychological well-being will be evaluated both before and after the program. The intervention is expected to be well-received, with high levels of satisfaction and engagement, leading to a greater awareness of healthy behaviors, improved psychological flexibility, and enhanced overall well-being. Demetra offers an accessible solution to support women through the transformative experience of motherhood with a multidisciplinary and innovative approach.

## 1. Introduction

### 1.1. Overview

It is becoming increasingly evident that lifestyles and exposure to risk or protective factors can profoundly impact health, interacting with genetic predisposition to determine medium- and long-term outcomes [1,2,3].

For instance, inadequate nutrition during pregnancy, lack of breastfeeding, or exposure to toxic substances and environmental pollutants may contribute to the development of metabolic disorders and obesity in adulthood. Awareness of these factors is critical, as they represent a public health priority [4]. Early intervention during pregnancy is essential, not only to mitigate future risks for both the mother and the infant but also as a unique opportunity to promote lifestyle improvements within the family, thereby establishing the foundation for long-term health.

Regarding weight, women who become pregnant while overweight or obese have a higher risk of complications during pregnancy and childbirth. These include the risk of impaired glucose tolerance, gestational diabetes, miscarriage, pre-eclampsia, thromboembolism, and maternal death. An obese woman is more likely to experience induced labor, prolonged labor, instrumental delivery, cesarean section, or postpartum hemorrhage; labor may also require higher doses of pain relief, which can be difficult to administer in obese conditions. There is also a greater likelihood of requiring general anesthesia, and after delivery, wound healing may be slower, with an increased risk of infection. Additional breastfeeding support may also be necessary due to challenges such as difficulty in latching the infant. Obese women may also have fewer options regarding where and how to give birth.

Children born to overweight or obese women also face various health risks. These include a higher probability of fetal death, stillbirth, congenital abnormalities, shoulder dystocia, macrosomia, and subsequent obesity [5,6]. The analysis of data available for the period 2015–2019 shows that the same situation applies to the population of women living in Trentino [7]. In fact, this study demonstrates that maternal excess weight and obesity are linked to a higher risk of gestational diabetes, gestational hypertension, pre-eclampsia, cesarean section, induction, postpartum bleeding, fetal macrosomia, and the need for neonatal resuscitation and NICU hospitalization. Additionally, infants born to overweight and obese mothers were less likely to be breastfed. Developing and implementing public health initiatives focused on preventing excessive weight gain during reproductive age could substantially improve both maternal and fetal health outcomes.

Cognitive Behavioral Therapy (CBT) techniques, particularly those related to third-wave therapies, have been shown to be more suitable than other psychotherapeutic approaches for being adapted into low-intensity interventions delivered through non-traditional methods such as e-health applications. This protocol, specifically, is based on the principles of Acceptance and Commitment Therapy (ACT), a third-wave form of CBT [8,9].

In developing this protocol, we conducted a thorough review of the scientific literature related to ACT in general and its application to individuals who are overweight or obese. ACT is a therapeutic approach based on two key concepts: acceptance (learning to accept thoughts by recognizing them as part of the human experience) and commitment (identifying what is important, one’s values, and taking action in that direction). It combines mindfulness practices with cognitive interventions aimed at increasing psychological flexibility—the ability to act effectively even in the presence of emotions or sensations perceived as negative [8,9].

The ACT approach proposes the use of a practical tool known as the matrix, which aims to help individuals visualize and better understand their feelings and act according to their values. The matrix is used throughout the intervention to facilitate awareness and psychological flexibility, as it helps individuals make choices based on their values rather than reactively responding to distress [2]. Regarding the application of the ACT model to overweight or obese individuals, a thorough review of several manuals was conducted, highlighting the potential of this approach for people with these physical characteristics. The adoption of the ACT model involves a reversal of the common paradigm, which includes a series of “quick fixes” or even “effortless weight loss”, focusing, instead, on providing space for the various processes that characterize the model: acceptance of negative thoughts, discomfort, and setbacks, not by fighting them but by learning to live with them; cognitive defusion from negative thoughts, which entails learning to distance oneself from invalidating thoughts; committed action, encouraging individuals to identify their values and act upon them; being in the present moment, through mindfulness, to increase focus on the present; self-compassion, learning to be kind to oneself in the face of potential mistakes. In summary, the literature review highlighted several studies that support the effectiveness of ACT in weight loss and weight reduction [10,11,12,13].

Therefore, the decision was made to divide this protocol into six sessions, each addressing one of the six core principles of the ACT model: values, committed action, acceptance, present moment contact, and self as context.

This study aims to design a digital health intervention through a virtual assistant (named Demetra and described in the following) available on mobile devices for providing women with initial information and suggestions about healthy habits during pregnancy, and then an ACT-based psychoeducational session to support the adoption of healthy lifestyles.

### 1.2. Local Background

The Autonomous Province of Trento (PAT) is committed to promoting community welfare by offering preventive, protective, and curative interventions dedicated to the whole pregnancy up to the first thousand days of the child’s life that are delivered through digital health tools [4].

In this context, the “Birth path-Dedicated midwife” is the set of services accompanying the birth event to guarantee information, education, counseling, and screening that is offered to promote and monitor the well-being of the mother, the newborn, and the family as a whole, and to promote the continuity of the care provided and pre-/post-natal assistance. The program is aimed at women who are pregnant, or who are trying to get pregnant, residing in the Province of Trento, and is provided according to the relevant territorial service. The timely care of the woman is guaranteed, and all necessary checks are scheduled from the beginning of the pregnancy to the end of the puerperium. According to the “Partnership caseload midwifery care” model, the midwife works in collaboration with the gynecologist, the general practitioner, the primary care pediatrician, and any other specialist involved in supporting the care before, during, and after childbirth based on the clinical condition of the woman. The Autonomous Province of Trento and the Provincial Healthcare Services (APSS) obtained the UNICEF-Baby Friendly Initiative accreditation in 2022, both for birth points and for the community. The birth point in Trento has been recognized as “Friends of Children” since 2015 [14,15,16].

The annual report on birth rates for the year 2023 [17] shows that 92.4% of all women giving birth in the Province of Trento joined the birth process described above. Taking into consideration the BMI data, 18.0% of women who became mothers were overweight, and 8.2% were obese. The probability of being obese before pregnancy among foreign mothers is almost twice as high as that of Italian mothers (14.3% vs. 6.4%), and this proportion is similar for overweight cases (28.1% vs. 15.0%). Moreover, the percentage of mothers in obese or overweight conditions increases with decreasing educational qualifications. Regarding obstetric and neonatal pregnancy outcomes, obesity is a risk factor for complicated pregnancies, in particular for pre-eclampsia and gestational diabetes. Also, the mode of birth is significantly associated with obesity risk; in fact, 24.8% of obese mothers have cesarean sections compared to 19.1% of mothers with normal weight. There is also a significant relationship between maternal obesity and weight at birth: 6.4% of newborns from obese women have a weight greater than or equal to 4000 g, compared to 4.7% of newborns from mothers with normal weight [17,18,19,20].

The research project described in this article fits into this context.

The project is supported by the TrentinoSalute4.0 Digital Health Program, and it is promoted by the Department of Health and Social Policies of the Autonomous Province of Trento, in collaboration with the Provincial Health Services Company (APSS) and under the technical–scientific management of the Bruno Kessler Foundation (FBK).

### 1.3. Goal of the Study and Research Questions

The present study is positioned within the design and development cycle of the Obesity-Related Behavioral Intervention Trials (ORBIT) model [21], specifically in phase 1a–1b of co-design and refinement of the intervention with key stakeholders and phase 2a of preliminary validation of its feasibility. The intervention consists of delivering educational content in text, audio, and video formats through a virtual coach (i.e., a digital assistant) named Demetra, integrated within the TreC platform. Participants will be able to log their food intake in a digital diary and receive personalized feedback from the system, as well as monitor their weight by entering weekly measurements. This intervention is grounded in the framework of behavior change pathways [22], aiming to support women in developing adaptive strategies to promote both physical and psychological well-being. It is important to clarify that, in this context, the term intervention refers to an application (specifically, the virtual coach) that provides evidence-based content at scheduled intervals, in a predefined, rule-based, structured, and systematic manner. All materials are reviewed by professional psychologists to ensure their quality and appropriateness. This material is available for users in audio, text, and/or video formats, and can be managed autonomously; therefore, the virtual coach presents itself as a tool for the delivery of information according to appropriate time frames. The intervention is developed for research purposes only, in order to promote the improvement of women’s physical and psychological well-being, by means of the modules based on the principles of ACT, as described in Section 2.1.

#### 1.3.1. Primary Objective

The primary objective is the co-design of the intervention with the involvement of domain experts and representatives of the target users (overweight pregnant women) from the beginning, to ensure the correspondence of the intervention to their requirements and needs. Moreover, we are going to perform an exploratory investigation of the experience and the engagement of women interacting with Demetra inside the TreC Mamma application. Semi-structured interviews will allow us to investigate how people felt during the intervention.

#### 1.3.2. Secondary Objectives

The secondary objective is to assess the level of physical and psychological well-being pre- and post-intervention through self-report questionnaires. Furthermore, the weekly monitoring of weight will allow a user to compare her own weight gain with her own growth curve for reference.

## 2. Materials and Methods

### 2.1. The Demetra Intervention

Demetra has a double purpose: at the beginning, it will provide information and educational tools for a healthy lifestyle during pregnancy and for gaining awareness of your real habits; after that, it will deliver a digital intervention based on ACT techniques for promoting healthy lifestyles through a psychoeducational approach. The intervention is targeted at pregnant women of legal age with overweight conditions and a good knowledge of both written and spoken Italian. As for technical skills, the only requirement is knowing how to download and use mobile apps.

Only an overview of the initial educational part will be given in this article, preferring to delve deeper into psychological ACT-based intervention. After a profiling data collection, pregnant women have at their disposal a microlearning course on healthy eating education, a 7-day food intake diary (for becoming aware of your real habits and receiving personalized feedback), a tool for monitoring the gaining weight in relation to the growth curves for pregnancy (both single and twin) and information, videos, and infographics about safe physical exercises organized by trimester of pregnancy. All these resources are presented and proposed at the beginning and remain available at all times. They were defined and prepared in collaboration with a nutrition biologist, while the physical exercises were developed with the support of a kinesiologist, both of whom were experienced in working with overweight pregnant women.

**Figure 1:**
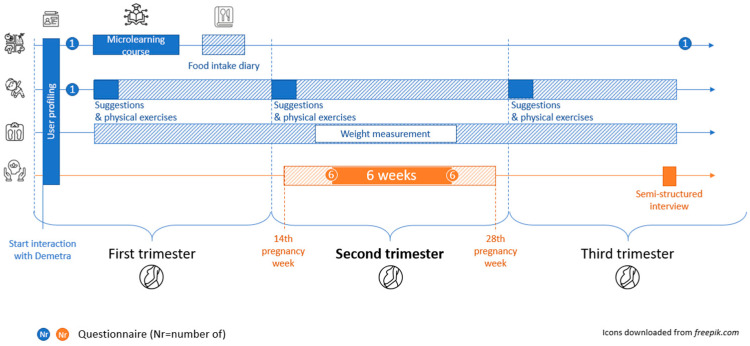
Overview of the intervention, including timing indications.

Going on with the next psychological part, Demetra provides ACT-based psychoeducational sessions through dialogs and multimedia materials (in video, audio, and image formats); it also plays the role of a virtual coach that guides women in carrying out exercises and tasks assigned to be carried out independently between one session and another. The acceptability and feasibility are going to be evaluated at the end of a pilot study (Figure 1).

The Demetra chatbot design was performed by a group of psychologists with specific expertise in communication of the Digital Health Research Unit (DHRes) of FBK and the Istituto Pavoniano Artigianelli of Trento, in collaboration with an experienced ACT psychotherapist. Afterwards, a revision by a Senior Psychologist of the Psychology Operating Unit of APSS was performed. All dialogs, videos, and audio tracks adopt an educational approach and do not follow an emergency management logic.

The psychoeducational intervention has a total duration of six weeks and includes one session per week, approximately 15 to 20 min in length. The goal of this intervention is to enable women to increase their psychological flexibility, allowing them to acquire and maintain healthy lifestyles despite experiencing negative emotions or feelings. The program content was developed by a team of clinical psychologists, in the absence of a pre-existing format, from ACT principles and a selection of reference texts, including Healthy Habits Suck [1], The Diet Trap [3], and The ACT Matrix [2].

Efforts will be made to achieve this purpose through a gradual delivery of the scheduled content. The women will also be asked to perform tasks independently and to fill in their personal diary available in the app on a daily basis, with the aim of promoting psychological flexibility and awareness with regard to eating behavior.

It is emphasized that this is not a device to replace in-person psychological support, should it be necessary. For this reason, the possibility of referral to a health professional is reminded throughout the various sessions where repeated malaise is manifested by women. A central aspect of the program is the deconstruction of dysfunctional beliefs related to weight control and weight loss, which are frequently present in the participants’ experiences. These issues are addressed through defusion strategies, experiences of contact with one’s own values, and acceptance practices, in line with the principles of ACT.

In the co-design phase of the intervention, two focus groups will be conducted involving health system professionals with various domain expertise and representatives of the target users. The objective is to verify the correspondence, completeness, and appropriateness of the mode of content presentation to the actual needs and preferences of the prospective users.

Each focus group will involve 6–10 representatives of the target figures and will be moderated by an FBK facilitator who will collect feedback from participants based on the presentation of some initial prototypes of the intervention.

The duration of each focus group will be approximately one hour. The data collected will then be analyzed mainly with qualitative methodologies, such as thematic analysis, and will allow for refining the digital modes of delivery of the intervention (without going to change the content of the intervention itself) to increase its acceptability and future usability and effectiveness. Participation in the focus groups will be on a voluntary basis and explicit consent will be sought through the informed consent form.

Following this phase, another co-design pilot study was planned, involving various domain experts (psychologists, communication experts, and reference clinicians) and some target users, to test the prototype and provide feedback and suggestions to improve the intervention itself before the start of the main study.

### 2.2. Study Design and Plan

This research project combines the principles of co-design, where stakeholders and final users actively participate in the design process, with a proof-of-concept design phase, to assess the feasibility and initial effectiveness of the designed digital intervention. This type of study requires a small, diverse, and representative sample (overweight pregnant women, healthcare professionals, and researchers) to ensure that the final service is well-designed, user-friendly, and meets the needs of the target audience.

In the psychological component of the intervention, the virtual coach Demetra will engage with participants for a continuous period of six weeks, spanning from the 14th to the 24th week of pregnancy. At the beginning of the program, six self-report questionnaires will be administered, as described in the Collected Parameters section, to establish baseline levels. The intervention will then be delivered through a variety of formats, including text, images, audio, and video. At the conclusion of the program, the same questionnaires will be re-administered to evaluate potential changes in eating habits, psychological well-being, and perceived quality of life.

Additionally, four more questionnaires will be administered to assess usability aspects, specifically user experience (UX) and user engagement (UE). All data collection procedures comply with current Italian regulations concerning personal data protection.

One month after completing the intervention, participants who have provided informed consent will be invited to take part in a semi-structured interview. This qualitative phase aims to explore their experience using Demetra within the TreC Mamma app and to gain insight into their emotional and cognitive responses throughout the process.

### 2.3. Participant Recruitment and Withdrawal

The target population of the study includes all overweight pregnant women residing in Trentino.

The inclusion criteria for participation in the study are as follows: (a) being pregnant; (b) being between the 14th and 24th week of gestation at the time of enrollment; (c) being aged 18 years or older; (d) owning a smartphone with internet access and the ability to download and use the application; (e) being a resident of the Autonomous Province of Trento; (f) having a good command of written and spoken Italian; (g) having a pre-pregnancy Body Mass Index (BMI) between 25.00 and 29.99; and (h) following an omnivorous diet. The exclusion criteria are as follows: (a) inability to provide informed consent (a prerequisite for study participation); (b) insufficient knowledge of the Italian language; (c) diagnosis of type 1 or type 2 diabetes mellitus (as these participants are already on a specific diet); (d) diagnosis of gestational diabetes in the current or any previous pregnancy; (e) fasting blood glucose levels between 100 and 125 mg/dL prior to or at the beginning of pregnancy; (f) belonging to populations with a high familial prevalence of diabetes (e.g., individuals from South Asia, the Caribbean, or the Middle East); (g) current or past diagnosis of an eating disorder; (h) being under the care of a dietitian; and (i) history of bariatric surgery.

Recruitment will take place among pregnant women belonging to the pregnancy care services of the APSS of Trento. Convenience sampling will be used. Midwives and/or doctors from the hospital and territorial services of the APSS will be involved in identifying women who are potentially eligible to participate in the study.

The study will begin after approval is granted by the Territorial Ethics Committee for Clinical Trials of the PAT.

Once approved, the timeline for the study is going to be as follows: the recruitment phase will take three months, followed by enrollment and the subsequent start of the study. There will be three months of follow-up. The study will end as data collection is concluded, no later than two months after the end of the pathway. Recruitment will go on until the planned sample size is reached.

### 2.4. Sample Size Estimate

It was estimated that for this proof-of-concept study, it will be necessary to recruit 50 volunteer pregnant women. This sample size was determined considering that both parametric and non-parametric statistics will be performed.

According to Noether [23], when assuming a non-normal distribution, non-parametric statistical analyses require a sample size of 24 pregnant women, considering the Bonferroni correction (α = 0.025), desired statistical power, and appropriate effect size for non-parametric tests such as the Kruskal–Wallis test and Wilcoxon post hoc test [24].

If parametric statistical analyses are also planned (assuming a normal distribution), a sample size of 41 pregnant women is required, accounting for the Bonferroni correction (α = 0.025), with a statistical power of 0.80 and a significance level of 0.025. Considering a potential dropout rate of 20%, it is estimated that a total of 50 pregnant women should be recruited for the study.

### 2.5. Technological Tools

The technological component of the study is based on the TreC platform, which enables citizens of the Trentino province to access, manage, and share health and well-being information [25]. TreC stands for ‘Cartella Clinica del Cittadino’ and is a reliable, well-tested platform designed to be a ‘system of systems’ rather than a simple data hub. A central pillar of the TreC platform is the citizen/patient’s role in managing their own health-related data. TreC’s flexible architecture enables the collection and management of heterogeneous data, as well as the development and use of additional subsystems that provide specific functions.

The digital intervention designed in this research project, in particular, is available in the TreC mobile application called ‘TreC Mamma’. It can be downloaded via a link only sent directly to participants. The user authentication process takes place via the digital identity service (Public Digital Identity System-SPID or Electronic Identity Card-CIE).

### 2.6. Procedures

#### 2.6.1. Enrollment

Women who meet the inclusion criteria and provide informed consent will be enrolled in the study. Participation requires signing an informed consent form at the time of enrollment, following a thorough explanation of the study objectives, data collection and management procedures, the expected level of involvement, the study duration, and issues related to confidentiality. Participants will also be informed that they may withdraw from the study at any time, without the need to provide a reason and without any impact on the quality of care received or on the continuation of their medical treatment.

In addition, participants will receive detailed information regarding the processing of their personal data during the study. Copies of both the informed consent form and the privacy policy will be provided to each participant and will remain accessible within the application throughout the study period.

#### 2.6.2. Data Collection

Socio-demographic data will be collected using a pre-structured form at the time of enrollment by the midwives and doctors involved. Each participant will be given a unique alphanumeric code. This socio-demographic data sheet will be kept separately from the data collected during the study. The required socio-demographic data will be: (a) date of birth, (b) expected date of delivery, (c) level of education, (d) occupation, (e) marital status, (f) number of previous pregnancies and deliveries, (g) partner’s occupation, if applicable, (h) pre-pregnancy weight, and (i) height.

Women will also be asked if they would like to participate in focus groups and qualitative interviews one month after the intervention ends (Table 1). If they consent, they will be asked for a contact telephone number.

The psychoeducational path offered by Demetra begins and ends with the completion of self-report questionnaires, which takes approximately 15 min.

Specifically, six questionnaires will be administered at the beginning and end of the psychoeducational intervention to investigate depressive and anxiety symptoms, general well-being, experiential avoidance, psychological flexibility, and adherence to the Mediterranean diet. None of the questionnaires had a diagnostic purpose, so they will not be used to diagnose psychopathology, but only for the collection of descriptive data.

A further four questionnaires will be sent out during the course with the aim of analyzing usability and the woman’s involvement with the app and the virtual coach, Demetra. Specifically, the usability questionnaires will be administered at the end of the third (three questionnaires) and sixth (end of the study; four questionnaires) weeks.

The choice of the ten assessment instruments was guided by the need to evaluate the feasibility, acceptability, and theoretical consistency of the Demetra intervention with the principles of Acceptance and Commitment Therapy (ACT). The selected tools cover three complementary domains:

1. Psychological variables—to detect changes in depressive and anxiety symptoms, overall psychological well-being, and psychological flexibility, which are directly relevant to ACT’s aim of increasing adaptive responses to internal experiences.
2. Behavioral and lifestyle outcomes—to measure adherence to healthy dietary patterns and physical activity levels, which are explicit behavioral targets of the intervention.
3. User experience and usability—to assess engagement, perceived quality, and usability of the app and virtual coach, which is crucial for evaluating the feasibility and future scalability of the digital intervention.

The Patient Health Questionnaire-9 (PHQ-9) and the Generalized Anxiety Disorder-7 (GAD-7) were chosen to measure depressive and anxiety symptoms, respectively, as these emotional states can interfere with the adoption of healthy behaviors and are relevant targets for ACT processes such as acceptance and cognitive defusion. The General Health Questionnaire-12 (GHQ-12) was included to provide a broader measure of general psychological well-being and functioning, complementing the PHQ-9 by assessing non-specific distress; the use of both tools allows for a more nuanced understanding of participants’ mental health status and distinguishes between general distress and specific depressive symptomatology. The Multidimensional Psychological Flexibility Inventory (MPFI) was selected as the primary theoretical outcome, as it directly assesses the six core processes of the ACT Hexaflex model, which the intervention aims to enhance. Two additional instruments, the MEDI-LITE and the International Physical Activity Questionnaire (IPAQ), assess adherence to the Mediterranean diet and physical activity levels, respectively, which are explicit behavioral targets of Demetra’s healthy lifestyle component and relate to the ACT process of committed action. The remaining four tools—User Engagement Scale—Short Form (UES-SF), System Usability Scale (SUS), Chatbot Usability Scale (BUS-11), and the User Mobile Application Rating Scale (uMARS)—were included to measure user engagement, usability, and perceived quality of the digital platform and the virtual coach. These usability and engagement metrics are critical in a feasibility study, as they inform the potential for sustained use and successful integration of ACT-based content into everyday life. This integrated battery of instruments allows for the simultaneous evaluation of psychological, behavioral, and technological engagement outcomes, providing a comprehensive picture of the feasibility and acceptability of the intervention.

An overview of the questionnaires and their administration schedule is provided in Table 2.

The Patient Health Questionnaire-9 (PHQ-9) [26] is used for the diagnosis, monitoring, and determination of depression severity. The PHQ-9 is recommended for both screening and case-finding and can be either self- or hetero-administered. It is an instrument consisting of two questions: the first one concerns the presence of the nine symptoms of depression (according to DSM-IV, also included in DSM-5), especially ‘in the last two weeks’. Through this question, the PHQ-9 score can be determined. Each symptom is assessed on a 4-point scale (0—never, 1—some days, 2—more than half of the days, and 3—almost every day); the second question assesses the functional understanding, caused by the depression, regarding the normal course of the patient’s life. The score of this question does not count towards the total score of the PHQ-9. The test score ranges from 0 to 27: between 5 and 9, depression is considered sub-threshold. A score of 10 is the optimal cut-off for showing clinically relevant depression [27] with three different levels of severity depending on the score [28].

The General Anxiety Disorder-7 (GAD-7) [29], validated in Italian by Pfizer-Italia Srl [25], is a useful self-report instrument for screening generalized anxiety disorder. Starting from the final score, based on cut-offs, it is possible to make a clinical categorization of: no severity, mild severity, moderate severity, or severe severity. It consists of four questions that can be answered using a 4-point scale (0—never, 1—some days, 2—more than half days, and 3—almost every day). Robust, non-parametric statistics were used to verify the one-dimensional nature of the GAD-7 [30].

The General Health Questionnaire (GHQ) [31] is a self-assessment instrument designed to identify mental health disorders and to monitor general psychological well-being. Developed by David Goldberg in 1970, the GHQ is widely used in clinical, research, and public health contexts. The 12 items of the questionnaire are formulated to investigate how the individual was feeling recently, with reference to psychological symptoms (such as anxiety and depression), ability to cope with everyday situations, sleep disturbances, and somatic symptoms. The response is based on a four-point Likert scale, ranging from “much better than usual” to “much worse than usual”, with a maximum score of 36.

The Multidimensional Psychological Flexibility Inventory (MPFI) [32] is an assessment instrument that measures psychological flexibility on several dimensions. It investigates how people adapt their thoughts and behavior in response to changing situations and challenges. The MPFI focuses on various aspects of psychological flexibility, including openness to experiences, present-moment awareness, and value-driven action. The 12 dimensions of flexibility and inflexibility (according to the Hexaflex model) are assessed using 60 Likert scale items ranging from 1 (“never true”) to 6 (“always true”). The Italian version of the scale was developed by Landi in 2021 [33].

A validated questionnaire, MEDI-LITE [34], gives an adherence score to the Mediterranean dietary pattern by assessing consumption of nine food groups. It assigns a numerical score (from 0 to 18), awarding more points for the high-frequency consumption of typical Mediterranean diet foods (e.g., fruit, vegetables, and pulses) and fewer points for the consumption of non-typical foods (e.g., meat and dairy products).

The International Physical Activity Questionnaire (IPAQ) [35,36] is a self-assessment tool that allows for measuring an individual’s level of physical activity. It classifies people into one of three categories (‘inactive’, ‘minimally active’, and ‘HEPA—health-enhancing physical activity—active’) based on the kinds of physical activities they do as part of their everyday lives and the time spent being physically active in the last 7 days. In this study, the short version was used, referring to pre-pregnancy physical activity.

The Chatbot Usability Scale (BUS-11) [37] is a tool designed to assess the usability, effectiveness, and satisfaction of users when interacting with chatbots. This instrument is structured around 11 items covering various aspects of the user experience with chatbots, aiming to provide an in-depth and multidimensional assessment of their usability. The items are answered on a 5-point Likert scale, where 1 equals “strongly disagree” and 5 equals “strongly agree”. The overall score ranges from 11 to 55.

The System Usability Scale (SUS) [38] is a quick and reliable tool for assessing the usability of systems. Developed by Brooke, it consists of 10 items and answers on a Likert scale of 1 to 5. The questionnaire provides an overall score that reflects the usability and applicability of a system or product. The score ranges from 10 to 50, depending on the degree of agreement with phrases such as ‘I found the system very easy to use’.

The User Engagement Scale—Short Form (UES-SF) [39] measures users’ engagement with digital technology through four subscales: focus, aesthetics, perceived interest, and perceived satisfaction. With only 12 items, it provides a quick but effective assessment of user engagement, resulting in scores that can range from 12 to 60 depending on the response given to the Likert scale ranging from 0 to 5, from ‘strongly agree’ to ‘strongly disagree’.

The User Mobile Application Rating Scale (uMARS) [40] is a tool to assess the quality of mobile applications. It consists of 26 items divided into 6 sections on engagement, functionality, aesthetics, information, subjective quality, and perceived impact. This allows for an in-depth and multidimensional assessment of the quality of mobile apps. Each item proposes answers on a Likert scale from 1 to 5, with 1 corresponding to ‘insufficient’ and 5 to ‘excellent’ [41].

### 2.7. Privacy and Data Protection

The study protocol was submitted to the Territorial Ethics Committee of the Autonomous Province of Trento (PAT) for clinical trials in the early months of 2025 and was approved under number A1061 of 05/03/2026.

At the time of enrollment, all women who voluntarily choose to participate will be asked to sign an informed consent form following a detailed explanation of the study objectives, expected level of involvement, study duration, and ethical aspects related to data confidentiality. Additionally, participants will be provided with a privacy statement outlining how personal data will be collected, managed, and processed, as well as the participants’ rights under applicable data-protection regulations.

Informed consent—including consent to the processing of special categories of personal data—must be given freely, voluntarily, explicitly, and in written form prior to participation. Copies of both the informed consent form and the privacy policy will be provided to each participant and will remain accessible within the app throughout the study.

Personal data will be processed solely for research purposes. The functionalities of the platform and the virtual coach are specifically designed to support the psychological well-being of pregnant women. All collected data will be treated as confidential; only data necessary for the evaluation of the study’s objectives will be processed, and these will be pseudo-anonymized prior to analysis.

The study coordinator is committed to preparing a comprehensive final report and to ensuring that all data are reported in a responsible and consistent manner. Personal data will not be disclosed or disseminated, except in anonymized or aggregated form. The results of the study will be published regardless of outcome, and dissemination through scientific publications, conference presentations, or seminars will occur exclusively in anonymized and aggregated formats that prevent any direct or indirect identification of participants.

The final results will be made publicly available one year after the conclusion of the study.

### 2.8. Data Analysis

#### 2.8.1. Quantitative Analysis

Statistical data processing will be conducted using R software, version 4.0.0 [42], IBM SPSS Statistics, version 27.0 [43], Stata 17 [44], and JASP, version 0.18.2 [45].

Categorical variables will be summarized using absolute and percentage frequency distributions, while quantitative variables will be described through appropriate measures of central tendency and variability.

Descriptive analyses will be conducted for both the physical–psychological variables (including depressive and anxiety symptoms, general well-being, experiential avoidance, psychological flexibility, physical activity, and eating habits) and the usability (UX) and user engagement (UE) variables. These analyses will be performed on data collected at the start, during, and at the conclusion of the interaction with Demetra.

The relationships between variables will primarily be examined using specific statistical tests such as the chi-square test, Fisher’s exact test, paired *t*-test, Wilcoxon’s non-parametric tests, and the sign test (depending on whether assumptions are met). This will help identify differences between baseline and post-intervention measurements within the same sample for the variables under study. Additionally, univariate logistic or multinomial regression models will be presented. To control for potential confounding variables, multivariate regression models will be employed, adjusting the effects of explanatory variables on outcome variables accordingly. A *p*-value ≤ 0.05 will be considered statistically significant for all analyses. Furthermore, McNemar’s test and Cochran’s Q test are planned for assessing paired qualitative data [46].

#### 2.8.2. Qualitative Analysis

Regarding the analysis of the semi-structured interviews, a text-mining approach will be employed to identify recurring responses related to women’s experiences interacting with Demetra and their feelings throughout the process. Additionally, qualitative methods such as thematic analysis will be applied to the focus group data.

Preliminary data analysis is scheduled for autumn 2025, with the final results expected at the study’s conclusion. The findings will be published within one year following the completion of the study

## 3. Expected Results

The psychoeducational pathway delivered through the virtual coach Demetra, integrated within the TreC Mamma app, is expected to have, first and foremost, significant results in terms of women’s satisfaction and engagement with the use of the innovative digital solution. Between the pre- and post-intervention period, it is expected to increase levels of physical and psychological well-being, women’s quality of life, and weight maintenance within their growth curve.

Importantly, Demetra can serve as a valuable resource by offering consistent psychoeducational support to women throughout and after pregnancy. Moreover, existing literature shows that women in the perinatal period often prefer online support, indicating that digital interventions can help overcome barriers related to social stigma and reluctance to seek help. Finally, it is essential to emphasize that Demetra and TreC Mamma are not intended to replace clinical or medical care but rather to complement these services by promoting physical and psychological well-being during pregnancy.

## 4. Discussion

This study aims to explore and assess women’s experiences and engagement with the TreC Mamma app and the virtual coach Demetra. Primarily, we seek to gather valuable feedback and suggestions from participants to enhance the intervention’s design, making it more engaging and improving user interaction. Additionally, we aim to observe potential differences in psychological well-being and healthy lifestyle behaviors before and after the intervention. However, we acknowledge that such changes may not be solely attributable to the intervention, and a randomized clinical trial will be conducted subsequently to rigorously evaluate its effectiveness.

All results will be reported and adequately discussed, including through a comparison with the relevant literature.

### Limitations

This study has several limitations that must be acknowledged and addressed when planning future studies. Firstly, as previously mentioned, it is important to note that the aim of this study is not to evaluate the effectiveness of the intervention; this is why a control group was not planned. In an RCT study, it will be crucial to involve a control group to assess actual effectiveness. Furthermore, the present study involved pregnant women from the Autonomous Province of Trento in Italy. Including participants from other Italian regions—and potentially other countries—would enhance the generalizability of the findings.

Secondly, the administration of multiple questionnaires may lead to participant overload, potentially affecting the quality of the collected data due to reduced motivation or rapid, unreflective responses. In addition, female participants will be recruited through hospital services by clinical staff without applying randomization or representative sampling methods, which may introduce a self-selection bias—participants may be more motivated or already familiar with digital technologies. Additionally, the follow-up period will cover only one month post-intervention, with no assessments during the postpartum phase, limiting the evaluation of long-term effects on lifestyle or psychological well-being. Lastly, although participants will be required to have sufficient knowledge of written and spoken Italian, their digital literacy will not be assessed. This may disproportionately affect women from certain social groups, potentially leading to early withdrawal from the study due to technical difficulties rather than lack of interest.

It is also important to highlight that Demetra can offer valuable technological support by providing regular psychoeducational services to women during pregnancy. Research has shown that women in the perinatal period often prefer online support, indicating that digital interventions can help overcome barriers such as social stigma and hesitation to seek help. Finally, it is essential to stress that these technological tools—Demetra and TreC Mamma—are not intended to replace clinical or medical care, but rather to complement them by supporting psychological and physical well-being throughout pregnancy.

## 5. Conclusions

The current literature emphasizes the increasing popularity of online support among women during the perinatal period and highlights the potential of digital interventions to overcome barriers related to social stigma and seeking help. Pregnancy is also an ideal time to focus on ourselves and find the motivation to change habits and improve lifestyle and physical activity. In this context, Demetra emerges as a promising resource, offering consistent psychoeducational support to help women change their habits and improve their lifestyle throughout pregnancy.

One crucial point to emphasize is that this protocol concerns a feasibility and usability study. If the results of this study are encouraging, as we expect, the next step will be to refine the intervention based on the feedback received during this study and design the next one, which, in our opinion, should be a controlled, randomized study to evaluate the effectiveness of the intervention. Once this step has also been completed, we can consider designing a real-world study to evaluate not only efficacy but also effectiveness.

A further step forward that will be essential to take will be to design the same intervention in different languages, making adjustments not only linguistically but also based on the culture of the participants.

## Figures and Tables

**Table 1:** Summary of the questions of the qualitative interview.

Interaction	How did you find the interaction with the chatbot? Were there enough alternatives among the answers to be given to the chatbot? Which feature of the interaction did you like the most? And the one you liked the least?
Content	Do you feel that the mode of communication (length of sentences, terms used) was appropriate to the content?
Engagement and consistency	Was communicating with the chatbot engaging? In other words, did it encourage you to participate?
General	Do you have any concerns? Do you have any criticisms? Do you think that overall the intervention is suitable for a pregnant woman? from 0 to 10? When do you think would be the ideal time to have this intervention (beginning, middle, or end of pregnancy)?

**Table 2:** Summary of the questionnaires administered and their timing.

At the Start of the Study (Week 0)	At the End of Week 3	At the End of the Study (Week 6)	Follow-Up (One Month After the End of the Intervention)
PHQ-9		PHQ-9	
GAD-7		GAD-7	
GHQ		GHQ	
MPFI		MPFI	
MEDI-LITE		MEDI-LITE	
IPAQ		IPAQ	
	UES-SF	UES-SF *	
	SUS	SUS *	
	ITA BUS B	ITA BUS B *	
		uMARS *	
			qualitative interview

Note. * These usability questionnaires will be administered the day after those investigating psychological variables, so as not to burden the participant during compilation.

## Data Availability

No new data were created or analyzed in this study.

## Abbreviations

The following abbreviations are used in this manuscript:

ACT	Acceptance and Committed Therapy
CBT	Cognitive Behavioral Therapy
PAT	Autonomous Province of Trento
APSS	Autonomous Province of Trento and the Provincial Healthcare Services
FBK	Bruno Kessler Foundation
ORBIT	The Obesity-Related Behavioral Intervention Trials
UX	User experience
UE	User engagement
BMI	Body mass index
SPID	Public Digital Identity System
CIE	Electronic identity card
PHQ-9	Patient Health Questionnaire-9
DSM-IV	Diagnostic and Statistical Manual of Mental Disorders, Fourth Edition
DSM-5	Diagnostic and Statistical Manual of Mental Disorders, Fifth Edition
GAD-7	General Anxiety Disorder-7
GHQ	General health questionnaire
MPFI	Multidimensional Psychological Flexibility Inventory
MEDI-LITE	Mediterranean Diet Adherence Questionnaire
IPAQ	International Physical Activity Questionnaire
BUS-11	Chatbot usability scale
SUS	System usability scale
UES-SF	User Engagement Scale—Short Form
uMARS	User Mobile Application Rating Scale
